# Solar Ultraviolet Irradiation Induces Decorin Degradation in Human Skin Likely via Neutrophil Elastase

**DOI:** 10.1371/journal.pone.0072563

**Published:** 2013-08-30

**Authors:** Yong Li, Wei Xia, Ying Liu, Henriette A. Remmer, John Voorhees, Gary J. Fisher

**Affiliations:** 1 Department of Dermatology, University of Michigan, Ann Arbor, Michigan, United States of America; 2 Department of Biological Chemistry, University of Michigan, Ann Arbor, Michigan, United States of America,; Illinois Institute of Technology, United States of America

## Abstract

Exposure of human skin to solar ultraviolet (UV) irradiation induces matrix metalloproteinase-1 (MMP-1) activity, which degrades type I collagen fibrils. Type I collagen is the most abundant protein in skin and constitutes the majority of skin connective tissue (dermis). Degradation of collagen fibrils impairs the structure and function of skin that characterize skin aging. Decorin is the predominant proteoglycan in human dermis. In model systems, decorin binds to and protects type I collagen fibrils from proteolytic degradation by enzymes such as MMP-1. Little is known regarding alterations of decorin in response to UV irradiation. We found that solar-simulated UV irradiation of human skin *in vivo* stimulated substantial decorin degradation, with kinetics similar to infiltration of polymorphonuclear (PMN) cells. Proteases that were released from isolated PMN cells degraded decorin *in vitro*. A highly selective inhibitor of neutrophil elastase blocked decorin breakdown by proteases released from PMN cells. Furthermore, purified neutrophil elastase cleaved decorin *in vitro* and generated fragments with similar molecular weights as those resulting from protease activity released from PMN cells, and as observed in UV-irradiated human skin. Cleavage of decorin by neutrophil elastase significantly augmented fragmentation of type I collagen fibrils by MMP-1. Taken together, these data indicate that PMN cell proteases, especially neutrophil elastase, degrade decorin, and this degradation renders collagen fibrils more susceptible to MMP-1 cleavage. These data identify decorin degradation and neutrophil elastase as potential therapeutic targets for mitigating sun exposure-induced collagen fibril degradation in human skin.

## Introduction

Type I collagen is the primary structural protein in dermal extracellular matrix (ECM). Solar ultraviolet (UV) irradiation induces proteolytic cleavage of type I collagen fibrils, which impairs skin structural integrity and function, and contributes to clinical features that are commonly observed in chronically sun-exposed skin [1], [2]. Acute UV irradiation dramatically increases *de novo* production of MMP-1 in skin resident cells [1], [2]. MMP-1 initiates collagen degradation by cleaving a single site near the C-terminal end of type I collagen fibrils; this cleavage creates characteristic “1/4” length and “3/4” length collagen fragments. Collagen fragments generated by MMP-1 undergo further degradation by other MMPs. MMP-1 activity is a key modulator of dermal ECM degradation and is thus a potential therapeutic target to lessen photodamage [3], [4].

In addition to inducing MMP-1 activity, UV irradiation rapidly induces influx of polymorphonuclear (PMN) cells, which contain a variety of proteases, such as neutrophil elastase, cathepsin G, and proteinase 3, in intracellular granules, which can be released into the ECM. The number of PMN cells is greatly increased within hours after UV irradiation and returns to baseline within 72 hours [5]–[7]. PMN cell proteases have multiple substrates and are involved in tissue damage during local inflammation [8]. However, the substrates and function of PMN cell proteases are not well-defined in the context of cutaneous photobiology.

Decorin is the predominant proteoglycan in human dermis [9]–[11]. Decorin functions through direct binding to functionally and structurally important proteins, such as cytokines, growth factors, and fibrillar collagen, in the dermal ECM [12], [13]. The interaction between decorin and type I collagen fibrils has been studied in detail [11], [14]–[16]. Decorin binds near the C-terminus of collagen fibrils and regulates fibrillogenesis and mechanical properties of collagen fibrils. Collagen fibrils intertwined with decorin provide a scaffold that plays a critical role in maintaining skin structural integrity and appropriate tensile strength. Decorin-deficient human diseases and decorin knock-out mice display defects in dermal collagen fibril structure characterized by thin and fragile skin [17], [18]. In addition, evidence suggests that binding of decorin in the same region as the MMP-1 cleavage site, inhibits collagen cleavage by MMP-1 [19]–[21].

Decorin exists in two major forms, glycanated and non-glycanated [14]. Human glycanated decorin is composed of a core protein containing 339 amino acids and an unbranched glycosaminoglycan (GAG) chain, which is covalently linked to the 4^th^ amino acid from the N-terminus of the core protein. Glycanated decorin varies greatly in size, ranging from 60 to 140 kilodaltons (kDa) as revealed by Western blot. This size variation is due to heterogeneity of GAG chains, which differ in composition and length [22], [23]. Non-glycanated decorin is comprised of the core protein, which contains oligosaccharide chains of varying lengths. In contrast to collagen, relatively little is known regarding the effects of UV irradiation on the levels and metabolism of decorin in human skin [24], [25].

The present study reveals rapid and substantial degradation of decorin following UV irradiation of human skin *in vivo*. Our data indicate that decorin degradation is mediated by PMN cell proteases and results in increased susceptibility of collagen to degradation by MMP-1.

## Results

### Solar UV Irradiation Stimulates Decorin Degradation in Human skin *in vivo*

**Figure 1:**
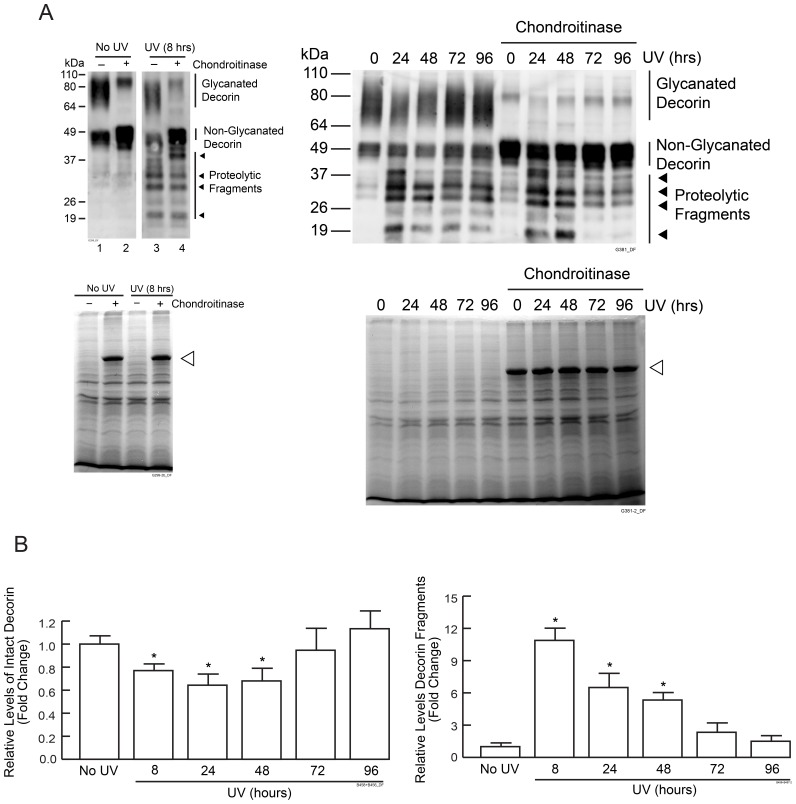
Solar-simulated UV irradiation induces decorin degradation in human skin *in vivo*. Human buttock skin was exposed to 2MED solar-simulated UV irradiation. Skin samples were obtained at indicated times and analyzed for decorin protein by Western blot. (A) Representative Western blots of native decorin, and decorin following removal of GAG obtained by chondroitinase. Four major decorin fragments (19–49 kDa) are indicated by arrow heads. 80 kDa bands in chondroitinase treated samples are homodimers of non-glycanated decorin. Coomassie blue staining of protein extracts are shown. Open arrowheads indicate bovine serum album, which is added in chondroitinase reactions. (B) Quantification of Western blots by ImageQuant software. Results are mean ± SEM, (N = 3–4; *p<0.05).

Sun-protected human buttock skin was exposed to two-times minimal erythema dose (MED) of solar-simulated UV irradiation, and full-thickness skin samples were obtained at baseline, 8 hours and 24 hour intervals for four days following UV irradiation (Fig. 1). Proteoglycans and other soluble proteins were extracted from skin samples and were analyzed by Western blot analysis using an antibody against C-terminal region of decorin core protein.

The presence of both glycanated and lower levels of non-glycanated decorin forms were observed (Fig. 1A). Glycanated forms varied between 70 kDa to 110 kDa. Non-glycanated decorin also appeared as several bands with molecular weights approximately 49 kDa, due to heterogeneity in length and composition of oligosaccharide chains [22].

Substantial reduction of both glycanated and non-glycanated decorin was observed 8 hours post UV irradiation and decorin levels remained reduced for 48 hours. Return to pre-UV exposure levels was observed between 72 and 96 hours post UV irradiation (Fig. 1B). Interestingly, concomitant with loss of decorin, there was the appearance of decorin fragments ranging in molecular weights between 19 kDa to 49 kDa. To quantify total decorin, protein extracts were treated with chondroitinase to remove glycan chains. Total decorin was reduced by nearly 25% at 8 hours and 40% at 24 hours post UV irradiation. Decorin fragments were increased by nearly 11-fold at 8 hours and 7-fold at 24 hours post UV irradiation. Chondroitinase treatment had no effect on the amount or size of decorin fragments as determined by Western blot, indicating that these fragments do not contain GAG chains. This finding is consistent with the glycan-linkage near the N-terminal and the antibody recognition-epitode near the C-terminal of the core protein. Four major fragments with approximate molecular weights of 19 kDa, 28 kDa, 32 kDa, 40 kDa, estimated based on Western blot, were observed (Fig. 1).

### Serine Proteases Released by PMN Cells Cleave Decorin *in vitro*

UV irradiation induces infiltration of PMN cells, which express multiple proteases [5]–[7], [26]. The time course of decorin fragmentation coincides with PMN cell infiltration [5], [7]. As such, we focused on the role of PMN cell proteases in UV irradiation-induced decorin degradation.

**Figure 2:**
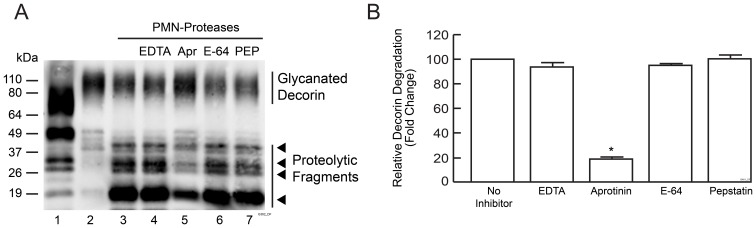
PMN cell released proteases cleave decorin *in vitro*. PMN cells were isolated from peripheral human blood and treated with vehicle or PMA. Conditioned media were collected and incubated with bovine decorin with or without protease inhibitors for 30 minutes. (A) Representative Western blot of decorin: Lane 1: partially degraded decorin in human skin 24 hours post UV irradiation. Lane 2: Bovine decorin-treated with conditioned media obtained from vehicle-treated PMN cells. Lanes 3–7: bovine decorin incubated with conditioned media obtained from PMA-treated cells with or without various protease inhibitors as indicated. EDTA is an inhibitor of metalloproteinase; Aprotinin (Apr) is an inhibitor of serine protease; E-64 is an inhibitor of cysteine protease; Pepstatin (PEP) is an inhibitor of aspartate protease. Four major decorin fragments are indicated by arrow heads. (b) Quantification of Western blots by ImageQuant software. Data are Western blots lanes 3–7. Results are mean ± SEM, (N = 3; *p<0.01).

To investigate the effect of PMN cell proteases on decorin, PMN cells were isolated from peripheral blood of healthy donors and treated with phorbol esters, which activate PMN cells to release proteases. Conditioned media containing released proteases were incubated with purified bovine decorin for 30 minutes. Bovine decorin core protein has 95% amino acid sequence homology to human decorin, although glycanated forms are larger than human decorin due to larger GAG chains. PMN cell proteases substantially degraded decorin, yielding four fragments with approximate molecular weights (19 kDa, 28 kDa, 32 kDa and 40 kDa), similar to those observed in UV-irradiated human skin (Fig. 2A, Lane 3 versus Lane 1), suggesting that PMN cell proteases contribute to decorin degradation in UV-irradiated skin.

PMN cells contain a variety of protease classes including serine, cysteine, aspartate, and metalloproteases. Inhibitors targeting each category of proteases were utilized to identify which classes of proteases are involved in decorin cleavage; these included metalloproteinase inhibitor EDTA, serine protease inhibitor aprotinin, cysteine protease inhibitor E-64, and aspartate protease inhibitor pepstatin. As shown in Figure 2A (Lanes 4–7), the serine protease inhibitor aprotinin largely blocked decorin proteolysis by 81%, while other protease inhibitors had no effect (Figs. 2A and 2B). These results suggest that serine proteases released by PMN cells cleave decorin.

### Neutrophil Elastase is the Predominant Decorin-degrading Protease Released by PMN Cells *in vitro*

**Figure 3:**
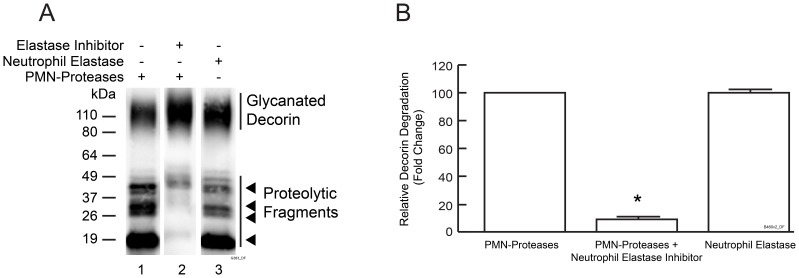
Neutrophil elastase cleaves decorin *in vitro*. Bovine decorin was incubated with conditioned media of PMA-treated PMN cells, or purified neutrophil elastase, with or without neutrophil elastase inhibitor (SSR69071) as indicated, and then analyzed by Western blot. (A) Representative Western blot. (B) Quantification of Western blots by ImageQuant software. Results are mean ± SEM, (N = 3; *p<0.01).

Neutrophil elastase is the major PMN cell serine protease [26] and was thus selected for further study. Purified human neutrophil elastase readily cleaved decorin (Fig. 3A, Lane 3) and generated four major fragments with molecular weights similar to fragments generated by PMN cell proteases. The highly selective neutrophil elastase inhibitor SSR69071 [27] largely inhibited decorin degradation by PMN proteases (reduced by 92%, Lanes 1 and 2 in Fig. 3A and Fig. 3B). These results indicate that neutrophil elastase is the predominant decorin-degrading PMN cell protease *in vitro*. Extension of incubation time with purified neutrophil elastase from 0.5 to 8 hours resulted in more complete decorin degradation, which generated decorin fragments too small to be detected by Western blot. Sequencing of these small fragments by Mass Spectrometry revealed multiple neutrophil elastase cleavage sites throughout decorin core protein (data not shown).

Taken together, the above data demonstrate that decorin is degraded by neutrophil elastase *in vitro*. Neutrophil elastase likely mediates UV irradiation induced decorin degradation in human skin *in vivo*.

### Decorin Binds to Collagen Fibrils and Inhibits MMP-1 Cleavage of Collagen

**Figure 4:**
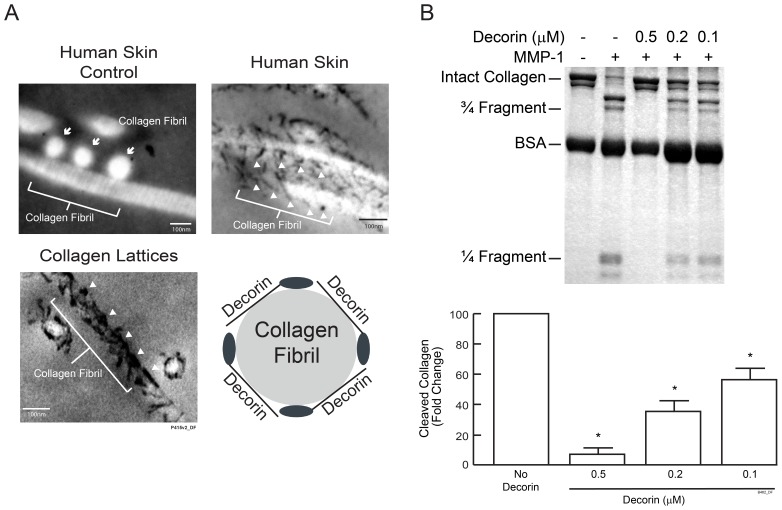
Decorin binds to collagen fibrils in human skin and inhibits MMP1 mediated collagen fibril cleavage. (A) Biopsies from normal buttock skin and collagen lattices containing decorin were stained with cupromeronic blue and analyzed by transmission electron microscopy. Electron micrographs revealed that decorin binds to the surface of collagen fibrils in human skin *in vivo* and in collagen lattices. Micrograph of human skin section without cupromeronic blue staining is included as negative control for decorin staining. Micrograph of non-stained control shows three cross-sectioned collagen fibrils displayed as white circles and indicated by arrows, and a longitudinal-sectioned collagen fibril displayed as a white filament. In micrographs of stained skin, decorin appears as small black filaments or solid black spheres (white arrow heads) that are localized on the surface of longitudinal-sectioned collagen fibrils. Micrograph of collagen lattices shows decorin (white arrow heads) associated with longitudinal-sectioned and cross-sectioned collagen fibrils. The schematic drawing shows decorin core proteins (solid black ellipses) and GAG chains (black lines) intertwine with a collagen fibril in a cross-section view. (B) Collagen lattices with or without decorin were treated with MMP-1. Collagen fragmentation was analyzed by SDS-PAGE eletrophoresis followed by Coomassie blue staining. Collagen fragments were quantified using ImageQuant software. Results are mean ± SEM (N = 3; *p<0.01). Inset shows a representative gel.

Electron microscopy revealed that decorin, displayed as small dark spheres or dark filaments, was localized on the surface of collagen fibrils in human skin and in three dimensional collagen lattices (Fig. 4A). We next investigated the ability of decorin to regulate collagen fibril cleavage by MMP-1. Collagen lattices made with and without decorin were treated with MMP-1, and collagen cleavage was examined by SDS-PAGE followed by Coomassie blue staining. Results showed that in the absence of decorin, MMP-1 cleaved collagen fibrils to yield characteristic fragments with sizes of “1/4” or “3/4” of intact collagen (Fig. 4B). Decorin inhibited MMP-1 cleavage of collagen fibrils in a dose dependent manner. Decorin (0.5 µM) reduced collagen fibril cleavage by more than 90% (Fig. 4B).

### Degradation of Decorin by Neutrophil Elastase Renders Collagen Fibrils more Susceptible for MMP-1 Cleavage *in vitro*

**Figure 5:**
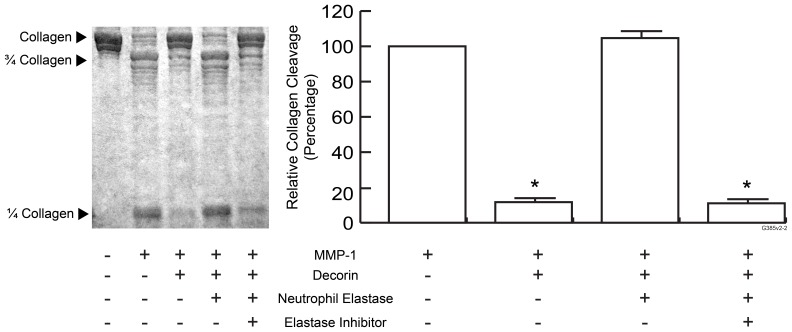
Degradation of decorin by neutrophil elastase increases cleavage of collagen fibrils by MMP-1. Collagen lattices with or without decorin were treated with or without MMP-1 and/or neutrophil elastase with or without neutrophil elastase inhibitor as indicated. Collagen fragmentation was analyzed by SDS-PAGE eletrophoresis followed by Coomassie blue staining. Collagen fragments were quantified using ImageQuant software. Data are Western blot lanes 2–5. Results are mean ± SEM (N = 3; *p<0.01). Inset shows a representative gel.

Based on inhibitory effect of decorin on MMP-1 cleavage of collagen fibrils, we hypothesized that neutrophil elastase-mediated degradation of decorin in human skin following UV irradiation enhances MMP-1-mediated cleavage of collagen fibrils. We tested this hypothesis using collagen lattices (Fig. 5). Decorin (0.5 µM) reduced MMP-1-catalyzed collagen cleavage by approximately 90%. This inhibitory effect was completely eliminated by addition of purified neutrophil elastase (0.01unit/ml). Neutrophil elastase inhibitor (SSR69071, 1 nM) protected decorin from proteolytic activity of neutrophil elastase and thus protected collagen cleavage from MMP-1. Neutrophil elastase alone did not cleave collagen fibrils (data not shown).

**Figure 6:**
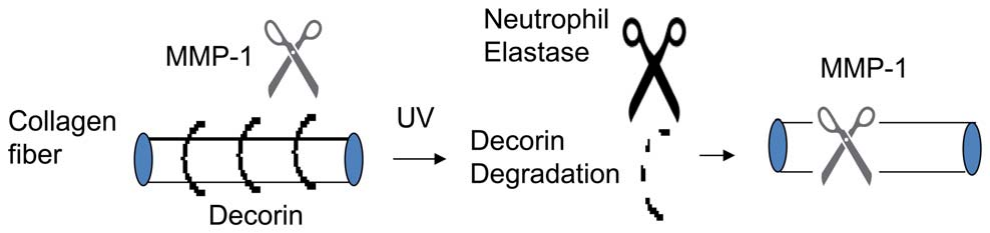
Solar ultraviolet irradiation induces decorin degradation in human skin via induction of neutrophil elastase. Decorin degradation by ultraviolet (UV)-induced neutrophil elastases increases susceptibility of type I collagen to cleavage by matrix metalloproteinase-1 (MMP-1).

In summary, our data indicate that decorin binds to collagen fibrils in human skin and collagen lattices, and protects collagen fibrils from cleavage by MMP-1. UV irradiation induces infiltration of PMN cells, which contain neutrophil elastase that can degrade decorin. Degradation of decorin renders collagen more susceptible to cleavage by MMP-1 (Fig. 6).

## Discussion

Decorin is a ubiquitous extracellular matrix protein and plays important physiological roles in connective tissues homeostasis [13]. In addition, decorin is also involved in various pathological processes, such as wound healing, fibrosis, inflammation and tumor invasion, in which remodeling of connective tissues occur [13], [28]–[33]. The multifunctional role of decorin is due to its capacity to modulate the activity and/or stability of a wide variety of proteins, such as cytokines, growth factors, cell surface receptors and structural matrix proteins via direct binding to these proteins. Impairment of decorin binding to its partners due to aberrant decorin degradation is linked to fibrotic wound healing [28]. Recent data suggest that granzyme B, a serine protease produced mainly by cytotoxic lymphocytes is capable of cleaving decorin [34], [35]. Proteases in skin that are responsible for decorin degradation during normal turnover or pathological conditions remain to be elucidated.

UV irradiation-induced MMP-1-mediated degradation of type I collagen fibrils significantly contributes to premature skin aging caused by chronic exposure to solar UV irradiation [1]. We found that decorin, the predominant collagen-binding proteoglycan in human dermis, undergoes rapid and substantial degradation in response to UV irradiation in human skin *in vivo*. This degradation is accompanied by accumulation of decorin fragments. Decorin degradation occurs within 8 hours post UV irradiation, which coincides with initial infiltration of PMN cells [5], [7]. PMN cells contain neutrophil elastase, which cleaves decorin and generates fragments with molecular weights similar to fragments observed in UV-irradiated skin. These data indicate that neutrophil elastase is a major protease responsible for decorin degradation. Binding of decorin to collagen fibrils protects them from MMP1-catalyzed cleavage and degradation of decorin collagen fibrils become more susceptible to cleavage by MMP-1[19]–[21]. The present data support a paradigm of collaborative and sequential actions of neutrophil elastase and MMP-1 on degradation of type I collagen fibrils. Future studies are needed to elucidate the role of proteases other than neutrophil elastase, such as granzyme B, MMPs and lysosomal proteases [34]–[37] and the fate of decorin GAG chains in UV irradiated skin.

Recent evidence suggests that decorin fragments function as proinflammatory signaling molecules [32]. Therefore, in addition to promoting collagen fibril degradation, breakdown of decorin and release of its fragments into extracellular microenvironment might play a role in UV irradiation-induced inflammation. A specific neutrophil elastase inhibitor might be used to protect decorin and consequently reduce inflammation and collagen fibril proteolysis following exposure of human skin to UV irradiation. Decorin degradation has been described in inflammatory diseases involving destruction of connective tissues, such as periodontitis and osteoarthritis [38]–[40]. Proteases responsible for decorin degradation in these diseases have not been identified. We speculate that neutrophil elastase may be responsible for decorin cleavage in inflammatory conditions characterized by neutrophil infiltration [8]. Consistent with this hypothesis, we have found substantial degradation of decorin, with accumulation of fragments with similar molecular weights as those generated by neutrophil elastase in psoriatic lesions (unpublished observation). Recent evidence suggests that granzyme B, a protease produced by T cells, natural killer cells and other cell types, cleaves decorin [34], [35]. Decorin fragments generated by granzyme B differ in molecular weight from those generated by neutrophil elastase, suggesting these two proteases cleave decorin at different sites.

A low level of decorin fragments are observed in normal sun-protected buttock skin (Fig. 1). Given that the sizes of decorin fragments appear similar in non-irradiated and UV-irradiated skin (Fig. 1), it is possible that a member(s) of the elastase family, which includes several functionally related proteases, produced by resident dermal cells might be responsible for physiological decorin turnover [8], [41].

Both granzyme B and neutrophil elastase are likely involved in inflammation associated decorin degradation as both proteases are produced by immune cells. Decorin cleavage by these two enzymes differs in several aspects. Decorin fragments generated by granzyme B differ in molecular weight from those generated by neutrophil elastase, suggesting these two proteases cleave decorin at different sites [34], [35]. The temporal and spatial expressions of these two proteases are likely different in a given inflammatory response, as neutrophils are much more abundant than cytotoxic lymphocytes in peripheral blood, and neutrophils influx into inflammatory lesions normally occurs prior to lymphocytes. Further characterization of decorin-degrading proteases would enhance our understanding of decorin biology and its involvement in diseases [13], [28]–[33].

## Materials and Methods

### Ethics Statement

This study was conducted in compliance with Declaration of Helsinki principles. All procedures involving human subjects were approved by the University of Michigan Institutional Review Board, and informed written consent was obtained from all human subjects.

### Reagent and Antibodies

Anti-decorin antibody, chondroitinase ABC, purified bovine decorin and purified neutrophil elastase were purchased from Sigma (St. Louis, MO). MMP-1 protein was purchased from Calbiochem (San Diego, CA). Rat tail type I collagen was purchased from BD Biosciences (Bedford, MA). Proteinase inhibitors E-64, pepstatin, and aprotinin were purchased from Roche Applied Science (Indianapolis, IN). EDTA (Disodium ethylenediaminetetraacetate) was purchased from Invitrogen (Carlsbad, CA), and elastase inhibitor (SSR69071) was purchased from Tocris Bioscience (Ellisville, MO).

### UV Irradiation and Human Tissue Procurement

Procurement of human samples was conducted at the Clinical Pharmacology Unit (CPU) within the Department of Dermatology, University of Michigan. This study included 4 women and 3 men, with age ranging from 23 to 55 (mean age is 37.5). Subjects had fair skin, falling into Fitzpatrick skin type categories I, II, and III.

Sun-protected buttock skin of healthy adult Caucasians was irradiated with twice minimum erythema dose (2MED) solar-simulated UV by using solar simulator (SPEC 450 W xenon arc solar simulator) as previously described [5], [42]. The UV spectrum was 0.00006% UVC, 6.6% UVB, 16.5% UVA2, and 76.8% UVA1. Light output was monitored with an IL1443 phototherapy radiometer and a SED240/UVB/UV photodetector (International Light, Newbury, MA).

Punch biopsies (4 mm in diameter) were taken at indicated time points after UV irradiation. Skin samples were immediately embedded in optimal cutting temperature compound (OCT, Tissue-Tek, Miles Inc., Elkhart, IN), snap frozen in liquid nitrogen, and stored at −86°C until sectioning.

### Proteoglycan Extraction and Western Blot Analysis

Dermal extracellular proteoglycans were extracted from skin samples as previously described [22]. Skin samples embedded in OCT were cut into 20-micron thick sections. Skin sections (total 500 microns) were washed in phosphate buffered saline (PBS) and immediately lysed in 300 µl Urea Lysis Buffer (50 mM Tris pH 7.4, Urea 7 M, 150 mM NaCl, and proteinase inhibitor cocktail (Roche)). Proteoglycan extracts were agitated at 4°C for 20 minutes and spun at 12,000 rpm for 10 minutes. Supernatants were taken and dialyzed against PBS overnight with two buffer exchanges. In some experiments, proteoglycan extracts were treated with chondroitinase ABC (0.2 units/ml) for 2 hours at 37°C. Protein concentrations were determined by Braford protein assay. Protein extracts (10 µg) were mixed with Laemmli loading buffer and subjected to SDS-PAGE electrophoresis followed by electrotransfer to PVDF membrane. Western blots were performed with anti-decorin antibody. The blots were developed with ECF chemifluorescence (GE Healthcare, NJ), scanned by STORM MolecularImager (Amersham Biosciences), and quantified using ImageQuant software.

### Cleavage of Decorin by Proteases Released from PMN Cells *in vitro*

Human polymorphonuclear (PMN) cells were isolated from the peripheral blood of healthy volunteers by modified Ficoll centrifugation as previously described [43]. PMN isolated from 10 ml blood were suspended in PBS (2 ml) containing DMSO vehicle, or PBS containing phorbol myristate acetate (PMA, 30 µM) for 30 minutes. PMA stimulates release of proteases from PMN cells. PMN cells were removed from conditioned PBS by centrifugation at 3,000 rpm for 2 minutes. Conditioned PBS (20 µl) was incubated with purified bovine decorin (1 µg) for 30 minutes at 37°C. For some experiments, conditioned PBS was pre-incubated with the following proteinase inhibitors, EDTA (10 mM), E-64 (5 µg/ml); Pepstatin (1 µg/ml); Aprotinin (10 µg/ml); and neutrophil elastase inhibitor SSR69071 (1 nM), for 20 minutes and then subsequently incubated with decorin. After incubation, decorin degradation was analyzed by SDS-PAGE and Western blot.

### Cleavage of Decorin by Purified Neutrophil Elastase

Purified human neutrophil elastase (0.01 unit/ml) was incubated with purified bovine decorin (1 µg) in PBS (20 µl) for 30 minutes at 37°C. Cleaved decorin was analyzed by Western blot.

### Electron Microscopy

Skin biopsies were taken from buttock skin of healthy volunteers (20–30 years old). Collagen lattices were made from acid-soluble rat tail type I collagen (1 mg/ml) with addition of decorin [44]. The GAG chains of decorin in skin samples and collagen lattices were stained by cupromeronic blue as previously described [45], [46]. Briefly, specimens were fixed using Fixation Solution (2.5% (w/v) glutaraldehyde, 25 mM acetic acid and 25 mM sodium acetate (pH 5.8) at 4°C for overnight. After fixation, specimens were washed by Wash Buffer I (25 mM acetic acid and 25 mM sodium acetate (pH 5.8)) and incubated with Staining Buffer (0.05% (w/v) Cupromeronic Blue, 0.1 M MgCl_2_ and 25 mM sodium acetate (pH 5.8), 2.5% glutaraldehyde (w/v)) for 2 hours at room temperature. Specimens were further incubated with 0.034 M Na_2_WO_4_ for one hour at room temperature. After staining, specimens were dehydrated in graded ethanol, embedded in LR white resins and cut into ultrathin sections. Sections were analyzed by transmission electron microscopy at the Microscopy Image Laboratory, University of Michigan.

### Cleavage of Type I Collagen Lattices by Purified MMP-1

MMP-1 proenzyme (Calbiochem) was activated by trypsin as previously described [44]. After activation trypsin was inhibited by addition of soybean trypsin inhibitor. Collagen lattices were incubated with activated MMP-1 (0.1 mg/ml) for 16 hours. In some experiments, before MMP-1 treatment, collagen lattices with decorin were treated with purified neutrophil elastases (0.01 unit/ml) for 24 hours at 37°C, or collagen lattices with decorin were treated with neutrophil elastase inhibitor SSR66071 (1 nM) for 30 minutes before treatment of neutrophil elastase. After MMP-1 treatment, collagen lattices were dissolved in Laemmi loading buffer and subjected to SDS-PAGE eletrophoresis. SDS-PAGE gels were stained by Coomassie blue. Collagen fragments were quantified by ImageQuant software.

### Statistical Analysis

Difference between controls and treatments were analyzed using paired, two-tailed t-test. Differences were considered statistically significant when p<0.05. Data are presented as mean ± SEM.
